# Inhalable *N*-Acetylcysteine-loaded Lactose-coated PLGA Nanoparticles for Tuberculosis Treatment

**DOI:** 10.1007/s11095-025-03889-1

**Published:** 2025-07-10

**Authors:** Kabi Raj Chaudhary, Cláudia Viegas, Paola Pirela, Mariana Atalaia, Beatriz Ruivinho, Sanchit Arora, Arti Singh, Pedro Brandão, Charan Singh, Pedro Fonte

**Affiliations:** 1 Department of Pharmaceutics, ISF College of Pharmacy, Moga, Affiliated to IK Gujral Punjab Technical University, Jalandhar, Punjab 142001 India; 2 Faculty of Medicine and Biomedical Sciences (FMCB), Universidade do Algarve, Gambelas Campus, 8005-139 Faro, Portugal; 3 Centro de Ciências do Mar do Algarve (CCMAR/CIMAR LA), Campus de Gambelas, Universidade do Algarve, 8005-139 Faro, Portugal; 4 iBB—Institute for Bioengineering and Biosciences, Department of Bioengineering, Instituto Superior Técnico, Universidade de Lisboa, Av Rovisco Pais, 1049-001 Lisboa, Portugal; 5 Associate Laboratory i4HB - Institute for Health and Bioeconomy at Instituto Superior Técnico, Universidade de Lisboa, Av Rovisco Pais, 1049-001 Lisboa, Portugal; 6 Department of Pharmaceutical Sciences, School of Health Science and Technology, UPES, Dehradun-248007, Uttarakhand India; 7 Egas Moniz Center for Interdisciplinary Research (CiiEM), Egas Moniz School of Health & Science, 2829-511 Almada, Portugal; 8 CQC-IMS, Department of Chemistry, University of Coimbra, Rua Larga, 3004-535 Coimbra, Portugal; 9 Department of Pharmaceutical Sciences, HNB Garhwal University (A Central University) Chauras Campus, 246174, Srinagar Garhwal, Uttrakhand India; 10 Department of Chemistry and Pharmacy, Faculty of Sciences and Technology, University of Algarve, Gambelas Campus, 8005-139 Faro, Portugal

**Keywords:** dry powder, inhalable particles, *N*-acetylcysteine, PLGA nanoparticles, pulmonary delivery, tuberculosis

## Abstract

**Objective:**

Glutathione (GSH), known for having mucolytic, anti-inflammatory, and antioxidant activities, is used in clinical practice in several pathologies, including tuberculosis (TB). *N*-acetylcysteine (NAC) has been primarily used to treat lung conditions and paracetamol-induced liver toxicity. However, NAC exhibits potential antimycobacterial activity through several mechanisms including immunomodulation, enhancement of GSH levels, and direct antimycobacterial effect. In this work, we aim to develop an effective drug delivery system for NAC for inhalable formulations.

**Methods:**

Herein, we report the development of lactose-coated NAC-loaded Poly(lactic-co-glycolic acid) (PLGA) nanoparticles (NAC-PLGA NPs) obtained by double emulsion methodology. Lactose has a double role, as a cryoprotectant agent and dispersant for inhalable formulations. The physicochemical properties of lactose-coated NAC-PLGA NPs were examined in terms of particle size, polydispersity index (PdI), zeta potential (ZP), encapsulation efficiency, and morphology. The *in vitro* release and lung deposition studies were assessed.

**Results:**

The physicochemical characterization studies revealed the compatibility of the drug with the selected excipients. Moreover, lactose-coated NAC-PLGA NPs showed particle size of 310 ± 3 nm, PdI of 0.15 ± 0.01, and of -11.5 ± 0.4 mV. The *in vitro* release study suggested a biphasic release profile. Likewise, *in vitro* lung deposition studies revealed desirable lung deposition parameters, indicating effective particle size for efficient pulmonary delivery. Additionally, *in vitro* studies for antimycobacterial activity exhibited superior antibacterial activity against *Mycobacterium Tuberculosis* (MTB) H37Rv.

**Conclusions:**

These preliminary findings suggest that lactose-coated NAC-PLGA NPs can open the door to new therapeutic options against one of the most drug-refractory and drug-resistant infectious diseases, TB.

## Introduction

Tuberculosis (TB) is one of the most contagious and deadliest diseases, emerging as a leading cause of mortality worldwide [1]. The contagious nature of TB is due to its etiological agent known as *Mycobacterium Tuberculosis* (*MTB)* [2]*.* According to the World Health Organization (WHO) Global TB Report 2023, a quarter of the total population has been infected with TB, with 7.5 million new infections reported globally in 2022 [3, 4]. Currently, TB is treated using a combination of first-line antibiotics, including isoniazid, rifampicin, pyrazinamide, and ethambutol, however for drug-resistant cases second-line agents are also used (e.g., fluoroquinolones, bedaquiline, delamanid) [5]. Although there are first and second-line approved drugs to fight TB, antibiotic resistance and undesirable side effects hinder the therapeutic outcomes. Current therapeutic options often require high-frequency dosing, leading to hepatotoxicity, ototoxicity, nephrotoxicity, neuropathy, and hypersensitivity, limiting treatment application [6–8]. Additionally, poor bioavailability caused by the hepatic metabolism of orally administered drugs decreases the efficacy of its systematic use. Similarly, the mutational behavior of mycobacterium, the complex nature of bacterial cell wall, and granuloma formation are important variables that cause drug resistance, including multiple drug resistance (MDR) and extensive drug resistance (XDR), leading to restrictions for its wider use and therapeutic failure [9, 10]. Poverty, unsanitary practices, and poor therapeutic compliance (including irregular and incomplete dosing) are other reasons that contribute to drug resistance [11, 12].

However, nanotechnology-inspired drug delivery systems exhibiting antimycobacterial activity are emerging as suitable pathways to overcome the afore mentioned constraints. Various drug nanocarriers such as NPs, including solid-lipid NPs (SLNs), nanostructured lipid carriers (NLCs), liposomes, polymeric particles or microparticles consisting of composites, metal–organic frameworks [13], and microspheres emerge as suitable drug development platforms for the treatment of TB [14–17].

*N-*acetylcysteine (NAC) is a U.S. Food and Drug Administration (US-FDA) approved drug and WHO-recommended essential list drug [18, 19]. The wide range of pharmacological activities of NAC makes it a highly investigated molecule. Among its various pharmacotherapeutic applications, the antimycobacterial activity of this drug is majorly investigated when administered in an inhalational route. NAC displays a dual mechanism of action, comprising direct antimicrobial action and suppression of the host oxidative response due to its antioxidative activity, and hence its interest in TB treatment. NAC is a precursor of glutathione (GSH) and facilitates the production of nitric oxide, which could bind with GSH to form *S-*nitroso glutathione (GSSNO), the major factor for the antimycobacterial effect [20, 21]. Interestingly, the thiol group of the molecule causes a redox imbalance of mycothiol (MSH) in the mycobacteria, which leads to inhibition of mycobacterial growth. In addition, NAC facilitates the production of cytokines, namely interleukin-2 (IL-2), interleukin-12 (IL-12), tumor necrosis factor (TNF), and interferon (IFN), by enhancing the immunological activities of various cells including natural killer (NK) cells and macrophages, which could further suppress the mycobacterial growth [22–24]. Despite being characterized by high solubility and high permeability, NAC exhibits poor bioavailability (~ 10%), which significantly limits its therapeutic efficacy. This challenge stems from its rapid metabolism and systemic clearance. In this sense, drug delivery systems that stabilize NAC, enhance its bioavailability, and provide targeted delivery while preventing premature degradation are fundamental. Thus, encapsulating NAC in polymeric NPs for pulmonary delivery via the inhalational route offers a promising solution. This approach allows for a localized increase in drug concentration at the site of action, minimizing systemic exposure and degradation, while improving therapeutic outcomes for respiratory conditions [25, 26].

Unfortunately, the complex nature of the airway anatomy of the lungs creates another challenge for drug delivery. The aerodynamic properties of inhalable particles greatly influence the drug deposition pattern to deep lung structures and can also determine that these particles will be immediately exhaled after inspiration [27, 28]. For instance, particles of micron size (1–5 µm) are regarded to be the most efficiently delivered into deep lungs, where mycobacteria predominate. Either nano range size particles (< 1 µm) delivery or larger micron size particles (> 5 µm) delivery showed inefficient drug deposition due to quick exhalation and deposition at the upper respiratory tract (trachea), respectively [29, 30]. In this regard, several researchers have demonstrated that microparticles with a particle size range of 1–5 µm are efficient dry powder formulation for deep lung targeting. Using biodegradable and biocompatible PLGA co-polymer for cargo delivery was approved by the US-FDA and European Medicines Agency (EMA) and provides a remarkable targeted capability with minimal or no toxicities [31–33].

Additionally, to address the challenge of achieving efficient drug delivery to the deep lung, excipients like lactose are often used as carriers in dry powder formulations. Lactose facilitates the formation of larger particle agglomerates, thereby increasing the aerodynamic diameter of the formulation. This strategy is particularly beneficial for improving the flowability and dispersion of smaller particles, such as nanoparticles, during inhalation. By creating microparticles within the ideal size range of 1–5 µm, lactose ensures optimal aerodynamic properties that prevent immediate exhalation while promoting efficient deposition in the deep lung structures. Furthermore, lactose is a biocompatible and does not elicit significant immune responses when deposited in the lungs (namely with alveolar macrophages), once it is readily metabolized by endogenous enzymes or cleared from the respiratory tract via mucociliary mechanisms. For these reasons, it is widely accepted excipient in inhalable formulations, enhancing the safety profile of the delivery system while maintaining its effectiveness [34–36].

Considering the pulmonary system, particularly the lungs, as the primary site of MTB infection, this study aims to develop an innovative formulation for the treatment of pulmonary TB via inhalation. The proposed dry powder formulation consists of lactose-coated NAC-PLGA NPs, designed to achieve controlled and sustained drug release. This strategy seeks to minimize dosing frequency, reduce systemic toxicity, improve patient compliance, ensure site-specific delivery, and enhance therapeutic efficacy.

## Materials and Methods

### Materials

NAC and polyvinyl alcohol (PVA) (PVA 78 K, 88% hydrolyzed grade) were purchased from TCI Chemical, India. PLGA (50:50 DL-PLG grade) and lactose (α-lactose monohydrate: InhaLac 160 of sieved grade) were obtained from Sigma Aldrich, New Delhi, India. Likewise, solvent dichloromethane (DCM) was provided by SRL Laboratory Pvt. Ltd, New Delhi, India. *MTB* H37Rv strain was purchased from TB Research Centre, Chennai, India. All the other chemicals used were of analytical grade.

### Preparation of NAC-PLGA NPs

In the experiment, three different formulations (F1, F2, and F3) were engineered by varying the drug: polymer ratio and PVA concentration to physicochemical characterization. Initially, 10 mg of the drug was dissolved in 1 mL of distilled water (W1: internal phase). In a separate vessel, 50 mg (F1), 100 mg (F2), or 150 mg (F3) of PLGA were dissolved in 3 mL of DCM (oil phase). Subsequently, the W1 phase was added to each of the respective oil phases under continuous stirring for 30 min to afford the primary W1/O emulsions, which were then subjected to probe sonication for 3 min using Sonics Vibra Cell (VCX 130 PB) USA. Each of the primary W1/O emulsions were then added dropwise to 5 mL PVA solution (W2: external phase) of three different concentrations: 0.25% w/v for F1, 0.50% w/v for F2 and 0.75% w/v for F3 to get W1/O/W2 double emulsion, followed by probe sonication for 2 min. Moreover, α-lactose monohydrate solution (cryoprotectant) equivalent to 2% w/v was further prepared in an aqueous solution. Afterward, developed W1/O/W2 double emulsions were slowly poured onto the cryoprotectant with continuous stirring for 24 h, to evaporate the organic solvent completely. Another batch was prepared in the same way by pouring the W1/O/W2 double emulsions into water. By the end of this process, the resulting organic solvent-free solutions were lyophilized at −30°C for 24 h, followed by freeze drying at 40°C for 48 h to obtain a dry powder of lactose-coated NAC-PLGA NPs and uncoated NAC-PLGA NPs, respectively [26, 37–40]. All the formulations were performed in triplicate.

### NPs Physicochemical Characterization

#### Particle Size, Polydispersity Index (PdI), and Zeta Potential (ZP) Measurement

The particle size of developed NAC-PLGA NPs was measured by dynamic light scattering (DLS) technique using Delsa Nano Zetasizer. Additionally, the polydispersity index (PdI) was measured to determine the stability of nanosuspension. Moreover, electrostatic repulsion between particles of the same charge, zeta potential (ZP), was measured by electrophoretic light scattering (ELS) technique using NanoZS/ZEN 3600 Zetasizer (Malvern instrument Ltd.UK). All samples were performed in triplicate in aliquots collected after solvent evaporation and before the drying process.

#### Entrapment Efficiency (EE), Drug Loading (DL), and Yield Recovery Determination

Entrapment efficiency (EE) percentage was determined by quantification of the free drug in the supernatant after centrifugation at 4500 rpm for 40 min (Centrifuge 5418R Eppendorf, USA). A UV visible spectrophotometer (UV-1700, Shimadzu, Japan) at λ_max_ 204 nm was used for free drug quantification. The formula to determine % EE is given below:

$$\%\mathrm{EE}=\frac{\mathrm{Total}\;\mathrm{added}\;\mathrm{drug}-\mathrm{Free}\;\mathrm{drug}\;\mathrm{in}\;\mathrm{supernatant}}{\mathrm{Total}\;\mathrm{added}\;\mathrm{drug}}\times100$$

Similarly, drug loading (DL) percentage was determined as the total amount of drug added to the formulation, divided by the total solid content added (drug + polymer), as given by the formula:

$$\%\mathrm{DL}=\frac{\mathrm{Total}\;\mathrm{added}\;\mathrm{drug}\;-\mathrm{Free}\;\mathrm{drug}\;\mathrm{in}\;\mathrm{supernatant}}{\mathrm{Total}\;\mathrm{drug}\;\mathrm{loaded}+\mathrm{polymer}}\times100$$

The relation between total powder mass recovered after freeze-drying and total solid content added to the formulation is required to determine yield (%) recovery as mentioned below:

$$\%\;\mathrm{Yield}=\frac{\mathrm{Total}\;\mathrm{powder}\;\mathrm{recovered}}{\mathrm{Total}\;\mathrm{solid}\;\mathrm{content}}\times100$$

All evaluations were performed in triplicate in aliquots collected after solvent evaporation and before the drying process.

#### Morphological Identification

Morphological characteristics of both uncoated NAC-PLGA and lactose-coated NAC-PLGA NPs were investigated using field-emission scanning electron microscopy (FE-SEM) of type JSM-5200 scanning microscope (JEOL Ltd, Tokyo, Japan) via sprinkled of powdered sample into SEM stub. Likewise, the internal structure of the developed formulation has also been determined with high-resolution transmission electron microscopy (HR-TEM). In addition, the coating of PLGA NPs was also confirmed through HR-TEM.

### Microparticles Physical-state Characterization Studies

#### Fourier-transform Infrared (FTIR) Spectroscopy

Drug and excipient interaction or compatibility can be identified by using FTIR spectroscopy (FTIR spectrometer of PerkinElmer, USA). Herein, with the help of infrared grade KBr, samples were finely ground and then pressed into pellets at ambient temperature followed by recording spectra over the range of 4000–400 cm^−1^. FTIR spectra for NAC, PLGA, physical mixture, and lactose-coated NAC-PLGA were recorded.

#### Differential Scanning Calorimetry (DSC)

Thermal properties of given samples for NAC, PLGA, physical mixture, and lactose-coated NAC-PLGA were determined by DSC analysis (Perkin Elmer, USA). Performing the analysis at a temperature ranging from 25°C to 300°C, a weighed amount of drug-loaded sealed aluminum pan was subjected to heating at the rate of 10°C/min under a nitrogen atmosphere purged at 40 mL/min.

#### Powder X-ray Diffraction (PXRD)

The crystalline nature of the given powder sample was assessed through wide-angle PXRD (X’PertPRO diffractometer, PANalytical, The Netherlands) at a voltage of 40kV and current 30 mA (range of 4° to 42°) using a step size of 0.02 2θ with a dwell time of 2 s. NAC, PLGA, physical mixture, and NAC-PLGA were examined for this study.

### Dry Powder Formulation Evaluation

#### Flow Property of Powder Determination

The flow property of freeze-dried NAC-PLGA NPs was studied through an angle of repose, Hausner ratio, and Carr’s index determination. First, an accurately weighed quantity of powder was poured into a graduated measuring cylinder of 10mL and the volume occupied was recorded as bulk volume (V_bulk_). The filled cylinder was then tapped 1000 times with the help of a tapped density tester (stamp volumeter STAV 2003: Engelmann Ludwigshafen Germany) and recorded as tapped volume (V_tap_). Finally, bulk density and tapped density were determined along with the Hausner ratio and Carr’s index using the given formula.

$$\mathrm{Hausner}\;\mathrm{ratio}=\frac{\mathrm{Tapped}\;\mathrm{density}}{\mathrm{Bulk}\;\mathrm{density}}$$

$$\%\mathrm{Carr}^\prime\mathrm s\;\mathrm{index}=\frac{\mathrm{Tapped}\;\mathrm{density}-\mathrm{Bulk}\;\mathrm{density}}{\mathrm{Tapped}\;\mathrm{density}}$$

#### *In Vitro *Dissolution Study

Release of NAC from freeze-dried lactose-coated NAC-PLGA and uncoated NAC-PLGA NPs was determined by an *in vitro* dissolution study using United States Pharmacopeia (USP) dissolution apparatus II. Maintaining the dissolution medium at pH 7.4 and temperature at 37.0 ± 0.5°C, an accurately weighted quantity of freeze-dried NAC-PLGA NPs (equivalent to 10 mg drug) were subjected to sprinkled on the surface of 300 mL PBS. Finally, the samples were withdrawn at a predefined time interval and subjected to spectrophotometric analysis at λ_max_ 204 nm [41].

#### *In Vitro *Pulmonary Deposition Study

The aerosolization properties of the developed formulation (freeze-dried lactose-coated NAC-PLGA and uncoated NAC-PLGA NPs) were studied using a next-generation impactor (NGI) (Copley Scientific, Nottingham, UK) equipped with a USP induction port (Apparatus 1, USP 35). An accurately weighed quantity (20 mg) of each formulation was placed in a gelatine capsule (size 3, Quali-V®, Qualicaps® Inc, Whitsett, USA), which was then inserted in a hand inhaler (Osmohaler®, Plastiape, Italy) followed by puncturing, with the airflow rate being maintained at 60 L/min, measured by a flow meter (DFM2, Copley Scientific, UK). Furthermore, aerosolization characteristics of powdered samples as mass median aerodynamic diameter (MMAD), geometric standard deviation (GSD), % fine particle fraction (FPF) and % emitted dose (ED) were determined. MMAD can be estimated as the particle distribution pattern in the lungs. GSD determines the variability in the diameter of inhalable particles in the system. FPF is the fraction of particles having an aerodynamic diameter below 5 µm of an administered dose. ED is the total amount of powdered formulation ejected from the gelatine capsule.

#### *In Vitro *Antimycobacterial Activity

Antimycobacterial activity of both free NAC and developed NAC-PLGA NPs were evaluated using the BACTEC technique for minimum inhibitory concentration (MIC) and minimum bactericidal concentration (MBC) against *MTB* H37Rv strain (TB Research Centre, Chennai, India). *MTB* H37Rv strains were cultured in a medium consisting of 10% oleic acid, albumin, and dextrose including Middlebrook 7H9 broth (OADC; HiMedia, India), which were subjected to aliquots at −65°C till required. BACTEC™ MGIT™ 960 System (Becton Dickinson, USA) was used to determine the MTB growth index (GI), over a period of 3–7 days, depending on the bacterial growth rate, for quantitative identification of ^14^CO_2_ released as expressed in a scale of 0 to 999 from ^14^C-labelled substrate metabolism in the medium. Herein, 0.1 mL bacterial culture inoculated BACTEC 12B vials were subjected to incubation at 37°C and 5% CO_2_ enriched medium, after incubation, MIC was recorded as the lowest concentration of the NAC that completely suppresses the growth. GI value was evaluated daily until and unless the values were observed to be greater than 30, under suitable aerobic condition of 1:100 control. Both positive and negative controls consisting of either bacterial suspension or broth alone were included in all test groups. The percentage of growth inhibition was estimated for each drug concentration. The determined MIC and MBC values provide strong evidence for growth inhibition activity and bactericidal potential of the developed formulations, respectively [26].

#### Statistical Analysis

All data are represented as mean ± standard deviation (SD) in triplicate. Student *t*-test was applied for comparative analysis of statistical data in the experiments performed using GraphPad Prism software.

## Results and Discussion

### Particle size, PdI, and ZP Determination

Among the three formulations developed in this study (F1-F3), the F2 formulation was selected for further characterization due to its optimal physicochemical properties, including a particle size of 310 ± 3 nm, a PdI of 0.15 ± 0.01, and a ZP of −11.5 ± 0.4 mV, as summarized in Table I. The F2 formulation, characterized by its desirable particle size, high %DL, and %EE, was identified as the optimized formulation for further investigation. In contrast, the particle sizes of the F1 and F3 formulations exceeded the acceptable range of 200–400 nm, rendering them unsuitable for further development. The increased size of the F1 NP compared to F2 is due to the aggregation of PLGA particles, as the PVA concentration is lower. Moreover, the F3 formulation has a larger particle size compared to F2 which might be due to the increased concentration of PLGA. These results are also justified by results reported by Manchanda *et al.* [42], showcasing an increment in particle size with increased PLGA concentration. Likewise, the lower PdI of the F2 formulation compared to F1 is due to the optimum PVA concentration that makes the nanosuspension more stable. PdI of F3 is higher than F2, which might be the reason for larger particle sizes leading to decreased stability of the nanosuspension. Increased negative ZP of F3 formulation is caused by higher PVA concentration. A similar effect of PVA concentration on particle size, PdI, and ZP was observed in various previous investigations reported in the literature [43, 44]. The particle size, PdI, and ZP of reconstituted freeze-dried lactose-coated NAC-PLGA NPs were found to be 320 ± 3 nm, 0.20 ± 0.10, and −11.8 ± 0.6 mV, respectively. The obtained results only showcase slight differences, indicating that our developed formulation, even after coating with lactose, remains stable.

**Table I:** Optimization of Three Different Formulations for F1, F2, and F3, Keeping the Drug Concentration Constant with Varying PLGA and PVA Concentrations

Formulations (NAC-PLGA)	Drug: Polymer ratio (w/w)	PVA conc (w/v)	Size (nm)	PdI	ZP (mV)	DL (%)	EE (%)
F1	1:5	0.25%	480 ± 6	0.42 ± 0.05	−8.3 ± 0.6	5.0 ± 0.2	47.0 ± 3.9
F2	1:10	0.50%	310 ± 3	0.15 ± 0.01	−11.5 ± 0.4	10.0 ± 0.2	72.0 ± 2.4
F3	1:15	0.75%	540 ± 7	0.31 ± 0.06	−12.6 ± 0.3	11.0 ± 1.0	77.0 ± 5.5

F2 formulation being a desirable particle size with good % DL and % EE was regarded as an optimized one and being further carried out for characterization

PdI: polydispersity index; DL: drug loading; EE: entrapment efficiency. Values are expressed as a mean ± standard deviation (SD), *n* = 3

### Drug Loading (DL), Entrapment Efficiency (EE), and Yield Recovery

As shown in Table I, the F2 formulation exhibits desirable particle size, PdI, and ZP with additional higher DL (10%) and EE (72%) when compared to the F1 formulation and with slight differences when compared with the F3 formulation. Some studies suggest that increased PVA concentration does not change the DL and, EE significantly, but can significantly impact particle size, PdI, and ZP [43, 44]. Moreover, the drug: PLGA ratio significantly changes the DL and EE. Increasing the PLGA concentration by keeping the drug concentration constant enhances the drug loading and entrapment efficiency as observed in the study. With the polymer content increasing while maintaining constant drug concentration, drug loading improves due to enhanced encapsulation efficiency and stronger polymer-drug interactions. A higher polymer concentration provides a larger matrix to retain the drug, reduces diffusion losses during formulation, and increases the viscosity of the polymer phase, which slows drug migration out of the system. Additionally, the greater availability of polymer minimizes competition for encapsulation, ensuring more drug is incorporated into the nanoparticles. This results in a denser, more efficient drug-polymer matrix, enhancing overall encapsulation efficiency and drug loading [45]. This is also in accordance with results previously reported in the literature [46, 47]. In addition, the % yield recovery of freeze-dried lactose-coated NAC-PLGA NPs was found to be 90%, while freeze-dried NAC-PLGA NPs was found to be 89%.

### Morphology Study by FE-SEM and HR-TEM

**Fig. 1:**
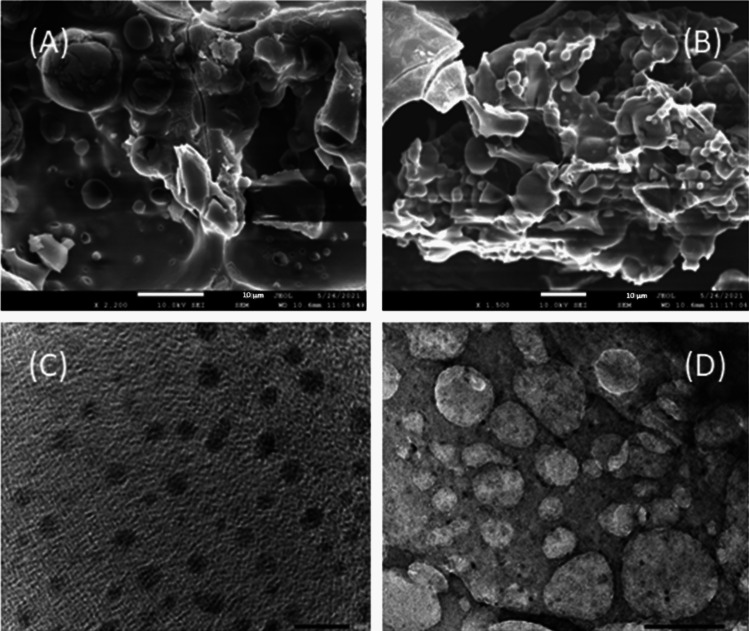
Morphological characterization of developed **(A)** uncoated NAC-PLGA NPs by FE-SEM, **(B)** lactose-coated NAC-PLGA NPs by FE-SEM, **(C)** uncoated NAC-PLGA NPs by HR-TEM, **(D)** lactose-coated NAC-PLGA NPs by HR-TEM. Bar scale represents 10 µm.

After morphological evaluation of uncoated as well as coated NAC-PLGA particles by FE-SEM, particles were observed as possessing spherical shape. Irrespective of uncoated NAC-PLGA NP, HR-TEM images confirmed that the lactose coating of PLGA particles enables the formation of inhalable PLGA composites. Figure 1 depicts **(A)** uncoated NAC-PLGA NPs by FE-SEM, **(B)** lactose-coated NAC-PLGA NPs by FE-SEM, **(C)** lactose-coated NAC-PLGA NPs by HR-TEM and **(D)** intensity distribution graph of uncoated NAC-PLGA NPs.

### FTIR

**Fig. 2:**
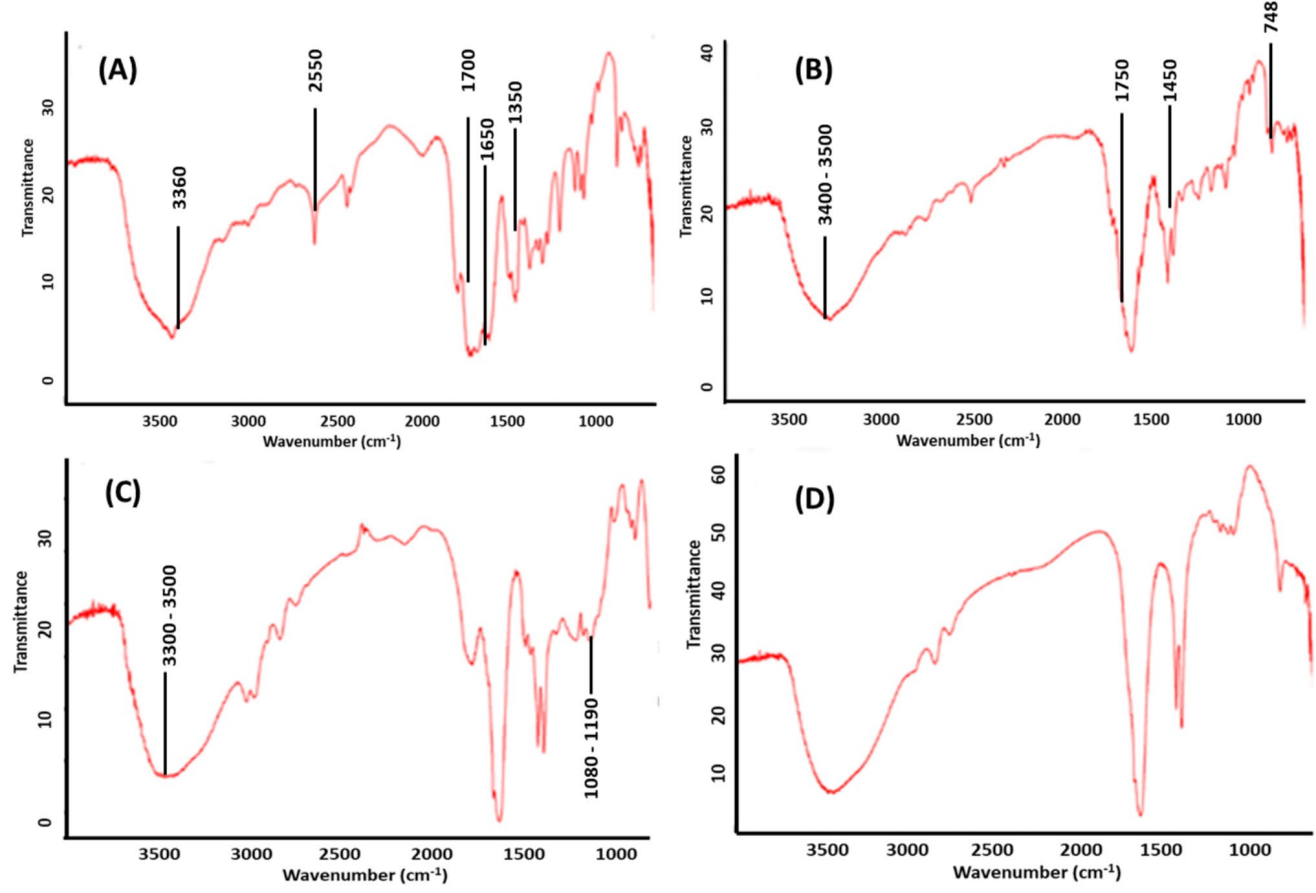
FTIR characterization of (**A**) NAC (drug), (**B**) PLGA (polymer), (**C**) NAC-PLGA-lactose (physical mixture), (**D**) lactose-coated NAC-PLGA NPs.

FTIR spectrum of NAC, PLGA, physical mixture of NAC-PLGA-lactose, and lactose-coated NAC-PLGA NPs were studied, finding compatibility between drug and excipients as shown in Fig. 2. FTIR spectra of NAC **(A)** observed at a different wavelength of 3360 cm^−1^, 2550 cm^−1^, 1700 cm^−1^, 1650 cm^−1^,1350 cm^−1^, 1080–1190 cm^−1^ is due to the presence of various functional groups characterized with stretching O–H, S–H group, C = O bond, COO bond, N–H group, and C-N groups of NAC, respectively. Moreover, ascribed peaks at a wavelength of 3400–3500 cm^−1^, 1750 cm^−1^, 1450 cm^−1^, and 748 cm^−1^ were observed in the FTIR spectrum of PLGA **(B)**, which was due to the presence of the O–H group, COO group, CH_3_ group, CH_2_ group, respectively. Henceforth, after observing the FTIR spectrum of the physical mixture **(C),** it was demonstrated that there is compatibility between the drug and excipients, with no significant changes in the mixture spectrum. Most importantly, the appearance of a broad band at 3300–3500 cm^−1^ with characteristics loss of S–H and C-N group’s peak at a wavelength of 2550 cm^−1^ and 1080–1190 cm^−1^ respectively in lactose-coated NAC-PLGA NPs, **(D)** suggested complete dispersion of NAC-PLGA NPs within the cryoprotectant. Our results were like those found in the literature [48–51].

### DSC

**Fig. 3:**
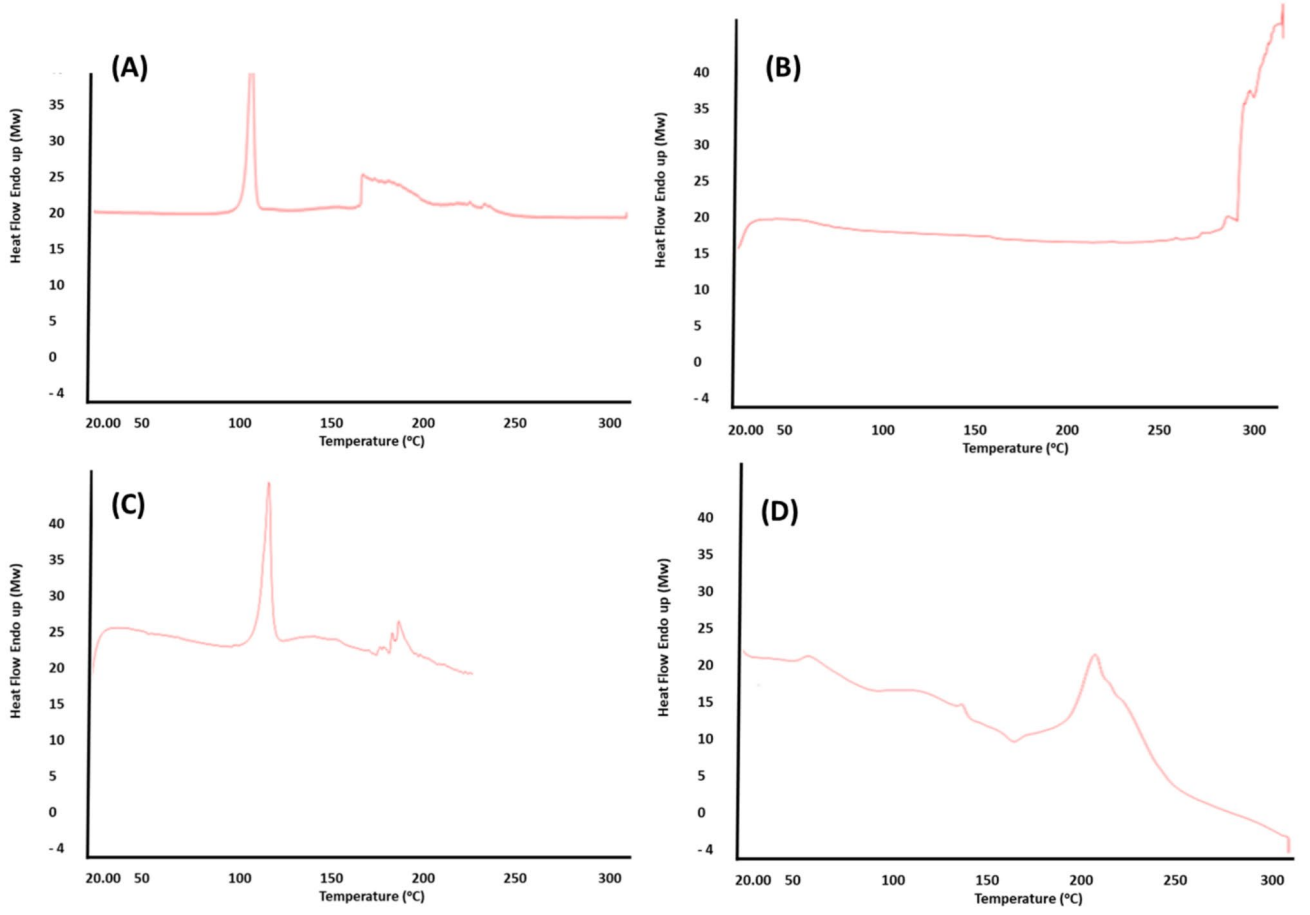
Thermal properties determination by DSC characterization at temperature up to 300°C of **(A)** NAC (Drug), **(B)** PLGA (polymer), **(C)** NAC-PLGA-Lactose (physical mixture), **(D)** lactose-coated NAC-PLGA NPs.

Thermal properties of NAC, PLGA, physical mixture of NAC-PLGA-lactose, and lactose-coated NAC-PLGA NPs were studied throughout the analysis of DSC thermogram as shown in Fig. 3. With exhibiting crystalline behavior, the obtained DSC thermogram for NAC **(A)** illustrated a sharp endothermic peak with a melting temperature (T_m_) at 111.95°C while the DSC thermogram of PLGA **(B)** showed glass transition temperature (Tg) at 50°C due to its amorphous characteristics. However, the DSC thermogram of the physical mixture of NAC-PLGA-lactose **(C)** exhibited the melting temperature (T_m_) of NAC at 110.30°C, Tg for PLGA at 55–60°C, and T_m_ for lactose was observed at a range of 160–175°C. The obtained results were aligned with the results reported by Mahumane *et al.* [49]. Finally, evaluating the DSC thermogram of lactose-coated NAC-PLGA NPs **(D),** it was observed that the intense peak of NAC was diminished along with the peak of PLGA, indicating that the NAC-PLGA NPs were significantly dispersed within the lactose dispersant matrix.

### PXRD

**Fig. 4:**
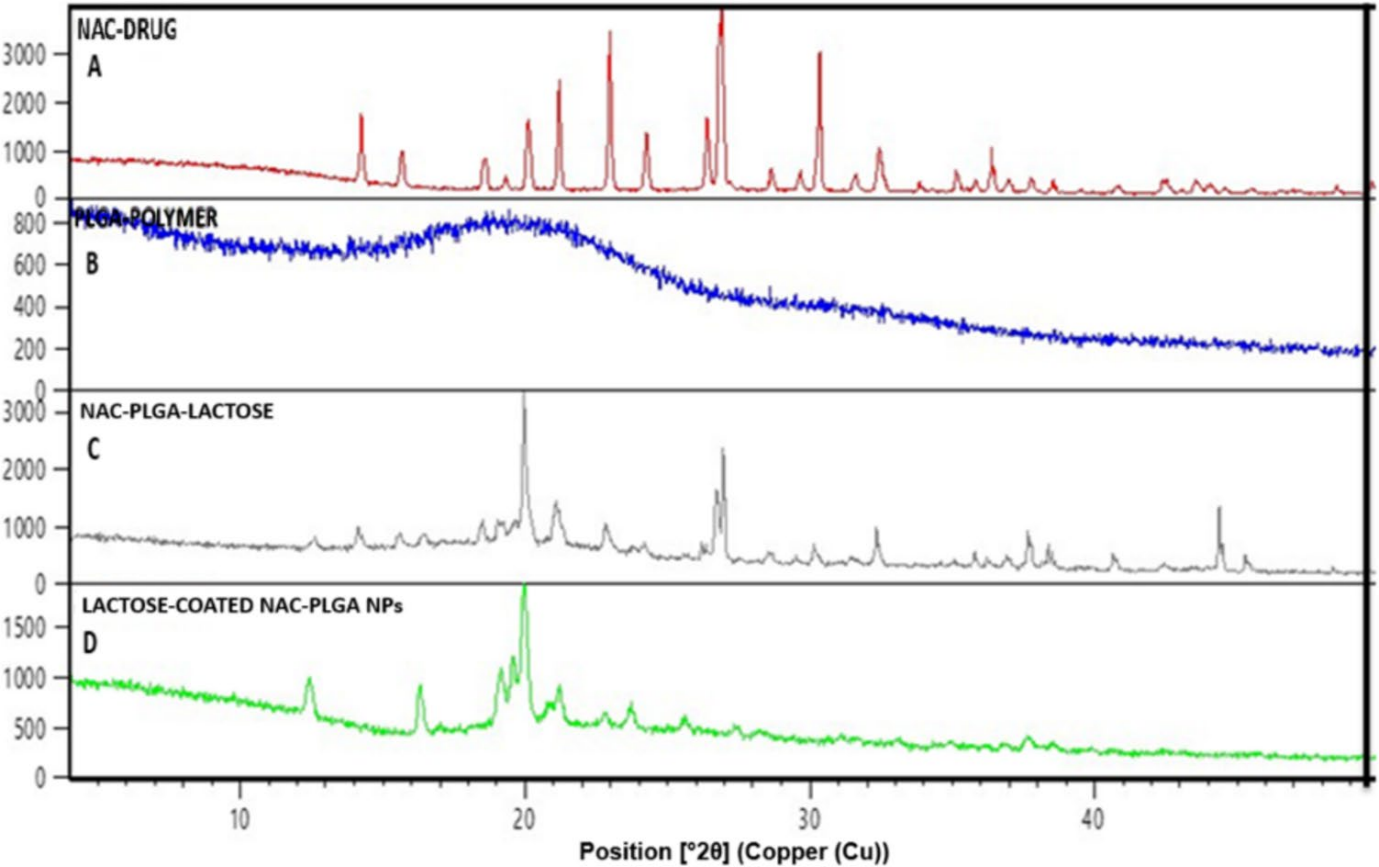
Powder x-ray diffraction (PXRD) of (**A**) NAC (drug), (**B**) PLGA (polymer), (**C**) NAC-PLGA-lactose (physical mixture), (**D**) lactose-coated NAC-PLGA NPs at position 2θ.

PXRD pattern of NAC, PLGA, and physical mixture of NAC-PLGA-lactose and lactose-coated NAC-PLGA NPs are depicted in Fig. 4. The sharp intense peaks of NAC **(A)** were visualized at 27^0^ followed by 21^0^, and 20^0^ at 2θ, suggesting the crystalline nature of the drug. In addition to this, a very broad pattern with a low intense peak of PLGA **(B)** at 19^0^ for 2θ, is depicted for its amorphous features. Moreover, each diffraction pattern for the respective NAC, PLGA, and lactose in the given physical mixture sample (NAC-PLGA-lactose) **(C)** was observed at its position in the given diffractogram, confirming compatibility between the drug and excipients. Importantly, the diffraction pattern of NAC was not observed in the final formulation of lactose-coated NAC-PLGA NPs **(D),** although a sharp intense peak of lactose was present at 20^0^ followed by small two peaks at 12^0^ and 16.5^0^ at 2θ, which concluded that NAC-PLGA particles were purely encapsulated within lactose cryoprotectant with no interaction between them. However, the results obtained in our study were satisfactory as compared to the Muhamane *et al.* findings [49].

### Flow Properties of the Powder

The angle of repose, Hausner ratio, and Carr’s index are indirect indicators to measure the powder flow properties. The flowability of a powder greatly influences the *in vitro* deposition [52]. In our study, the flowability of uncoated NAC-PLGA particles and lactose-coated freeze-dried NAC-PLGA NPs were investigated. The obtained values are listed in Table II. The angle of repose, Hausner ratio, and Carr’s index for lactose-coated NAC-PLGA NPs, indicate improved flow properties, whereas NAC-PLGA particles demonstrated very poor flowability. The reason for the undesirable flowability of uncoated particles was due to particle–particle aggregation [53]. Herein, the lactose's role as a formulation dispersant prevents particles from aggregating, hence enhancing the flowability.

**Table II:** Powder Flow Property of Coated (lactose-coated NAC-PLGA NPs) and Uncoated (NAC-PLGA NPs) Particles

Formulations	Angle of repose (θ)^a^	Bulk density (g/cm^3^)	Tapped density (g/cm^3^)	Carr’s index^b^	Hausner ratio^c^
Lactose-coated NAC-PLGA NPs	34.58 ± 1.20	0.152 ± 0.008	0.186 ± 0.004	18.27 ± 3.87	1.22 ± 6.04
NAC-PLGA NPs	62.16 ± 4.06	0.092 ± 0.004	0.150 ± 0.080	38.66 ± 5.20	1.63 ± 0.44

^a^Angle of Repose: 25–30, Excellent; 31–35, good; 36–40, fair; 41–45, passable; 46–55, poor; 56–65, very poor; > 66, very-very poor

^b^Carr’s Index: 5–12%, Excellent; 12–18%, good; 18–21%, fair; 21–25%, poor, fluid; 25–32%, poor, cohesive; 32–38%, very poor; > 40%, extremely poor

^c^Hausner ratio: 1.00–1.11, Excellent; 1.12–1.18, good; 1.19–1.25, fair; 1.26–1.34, passable; 1.35–1.45, poor; 1.46–159, very poor; > 1.60, very-very poor

### *In Vitro *Drug Release Study

**Fig. 5:**
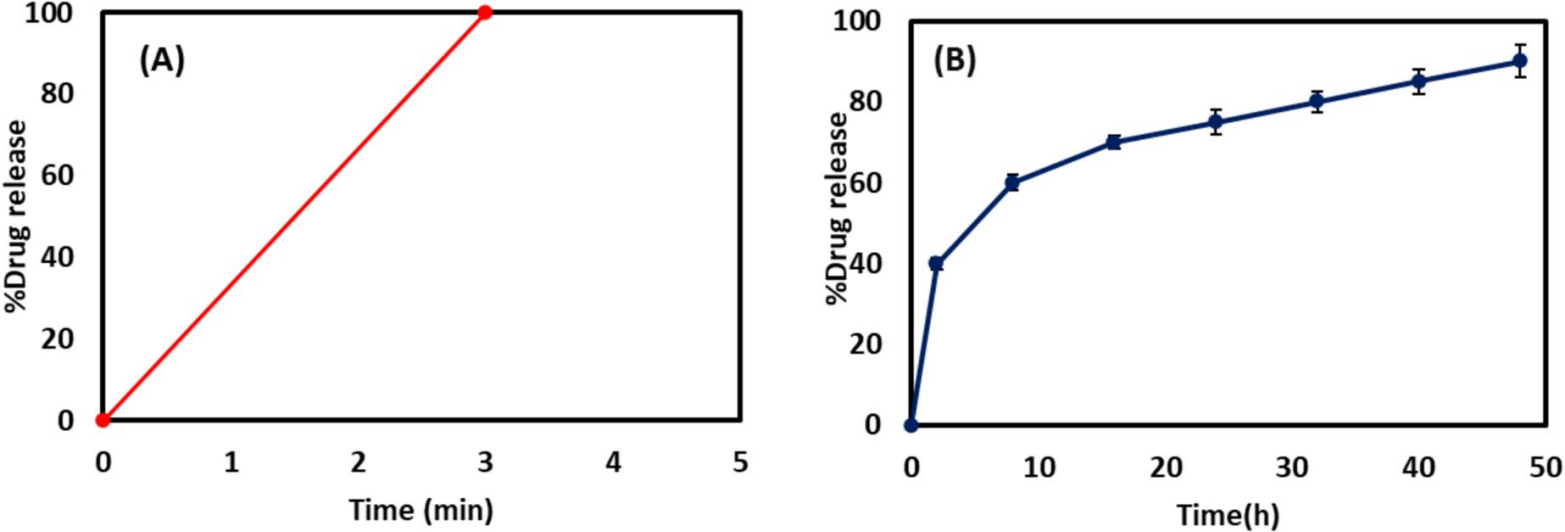
*In vitro* drug release profile of (**A**) free drug (NAC), (**B**) lactose-coated NAC-PLGA NPs at phosphate buffer of pH 7.4.

*In vitro,* release studies for lactose-coated NAC-PLGA NPs in phosphate buffer (at pH 7.4) indicated a biphasic pattern of release as depicted in Fig. 5(B). The initial burst release of 40% for two hours was visualized for this formulation due to rapid dissolution and release of adsorbed NAC at the surface of the particle or encapsulated near the particle surface. However, a slow, and sustained release profile was attained after 48 h with 90% drug release, indicating slow drug diffusion and matrix erosion once the PLGA NPs contact with the dissolution media and as the PLGA core degrades, NAC molecules also diffuse through the porous network formed by the degrading polymer. Additionally, the drug dissolution profile of free NAC was estimated to be 100% within 3 min as shown in Fig. 5(A), indicating the hydrophilic nature (solubility: 100 mg/mL) of NAC, as enlisted in BCS class I [54–56]. We need to take into account that drug release from biodegradable and biocompatible PLGA particles is highly dependent on the drug’s physical–chemical properties since drugs exhibiting low molecular weight and high hydrophilicity tend to display rapid leakage from polymeric particles [57–59].

### *In Vitro *Pulmonary Deposition Study

**Fig. 6:**
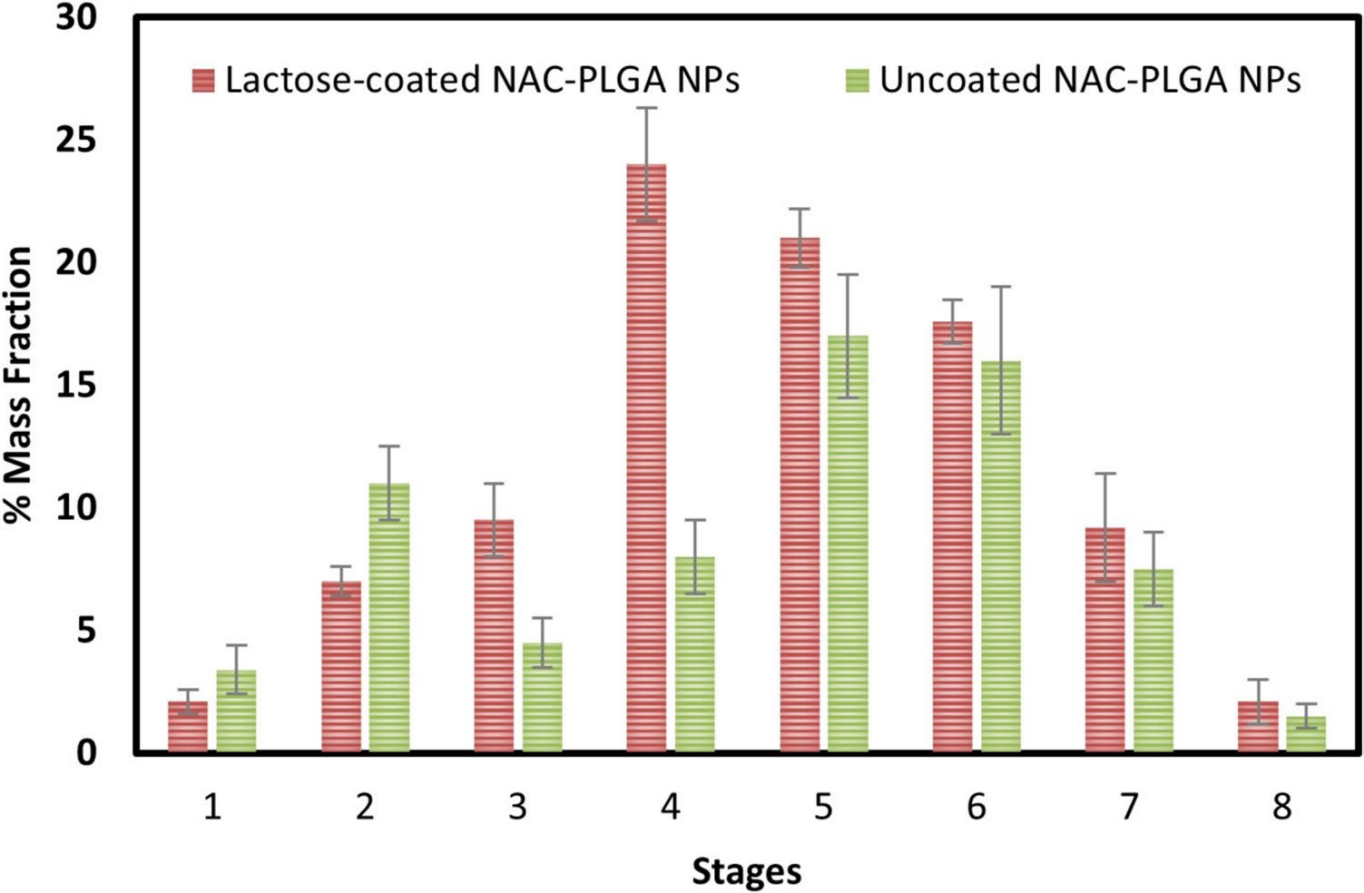
*In vitro* drug deposition study performed by Next Generation Impactor (NGI) of both uncoated NAC-PLGA NPs and lactose-coated NAC-PLGA NPs.

For the efficient delivery of dry powder via the inhalation route, its aerodynamic properties should be taken into consideration. Some studies revealed that MMAD ranging from 1 to 5 µm would be optimal for pulmonary delivery [60]. In our study, obtained results suggested that developed formulation showed an acceptable range of MMAD (2.50 ± 0.20 µm), GSD (1.49 ± 0.25 µm), FPF (65.50 ± 5.50%),and ED (92.28 ± 0.2%) as shown in Table III, indicating desirable features for an efficient deep pulmonary drug delivery [61, 62]. Moreover, the drug deposition study performed by NGI showed the highest deposition for lactose-coated NAC-PLGA NPs as compared to uncoated NAC-PLGA NPs at stage 3 followed by 4 and 5, indicating the deep lung deposition of the developed lactose-coated NPs, as depicted in Fig. 6. Singh *et al.* also observed the similar deposition of inhalable liposphere at stage 3 followed by 4 in a previously reported study [63].

**Table III:** *In Vitro* Aerosolization Study of Freeze-dried Lactose-coated (NAC-PLGA NCs) and Uncoated (NAC-PLGA NPs) Particles

Formulation	MMAD (µm)	GSD (µm)	FPF (%)	ED (%)
Lactose-coated NAC-PLGA NPs	2.50 ± 0.20	1.49 ± 0.25	65.50 ± 5.50	92.28 ± 0.20
Uncoated NAC-PLGA NPs	6.30 ± 0.40	1.68 ± 0.35	28.60 ± 5.50	68.43 ± 0.40

MMAD: mass median aerodynamic diameter; GSD: geometric standard deviation; FPF: fine particle fraction; ED: emitted dose. Values are expressed as a mean ± standard deviation (SD), *n* = 3

### *In Vitro* Study for Antimycobacterial Activity

The potency for the antimycobacterial activity of free NAC and developed freeze-dried lactose-coated NAC-PLGA NPs were investigated against the *MTB* H37Rv strain. MIC and MBC values were determined with 2.37 mg/mL and 19.25 mg/mL for free NAC, while 0.64 mg/mL and 5.18 mg/mL for the developed formulation, respectively. The inhibitory potential of NAC against mycobacterial strain were studied by various researchers, showing its potential to inhibit the growth of mycobacterium. In our study, freeze-dried lactose-coated NAC-PLGA NPs demonstrated a 4-fold increase in antimycobacterial activity, compared to free NAC [26, 64]. These results show that incorporating NAC in PLGA NPs coated with lactose as a cryoprotectant/dispersant agent can be a promising formulation strategy for pulmonary TB therapeutics.

## Conclusion

NAC-loaded PLGA particles were prepared by double emulsion method and were then coated with lactose followed by freeze-drying to obtain respirable NPs for deep-lung drug administration. The obtained results showed the compatibility between drug and excipients after physicochemical characterization, prolonged and sustained release of drug through *in vitro* release study, and efficient drug deposition to lower respiratory zone with an optimum MMAD (1–5 µm), establishing a promising formulation strategy for TB treatment. Importantly, the results obtained, showing higher *in vitro* drug deposition at stage 3 followed by 4 may significantly reduce the dosing frequency, comprising several therapeutic advantages, such as improved patient compliance and increased drug efficacy. Moreover, the biphasic release pattern with a slow and sustained release profile may further contribute to shutout rapid dosing.

Finally, freeze-dried lactose-coated NAC-PLGA nanoparticles exhibited a 4-fold enhancement in antimycobacterial activity compared to free NAC. However, further studies are required to comprehensively assess their *in vivo* antibacterial efficacy, including evaluating activity at multiple time points to determine both short- and long-term effects. It is also critical to evaluate whether the dose delivered locally to the lungs exceeds therapeutic levels, potentially leading to off-target effects such as altered immune cell activity, oxidative imbalance, or unintended cytotoxicity. Thus, the results herein reported pave the road to new formulation strategies towards a better approach for pulmonary TB treatment.
